# Functional cooperation of of IL-1β and RGS4 in the brachial plexus avulsion mediated brain reorganization

**DOI:** 10.1186/1749-7221-5-18

**Published:** 2010-12-07

**Authors:** Jifeng Li, Hui Zhao, Pengbo Luo, Yudong Gu

**Affiliations:** 1 Lab of Hand function reconstruction, Huashan Hospital, Fudan University, Shanghai, China

## Abstract

**Backgrounds:**

There is considerable evidence that central nervous system is continuously modulated by activity, behavior and skill acquisition. This study is to examine the reorganization in cortical and subcortical regions in response to brachial plexus avulsion.

**Methods:**

Adult C57BL/6 mice were divided into four groups: control, 1, 3 and 6 month of brachial plexus avulsion. IL-1β, IL-6 and RGS4 expression in cortex, brainstem and spinal cord were detected by BiostarM-140 s microarray and real-time PCR. RGS4 subcellular distribution and modulation were further analyzed by primary neuron culture and Western Blot.

**Results:**

After 1, 3 and 6 months of brachial plexus avulsion, 49 (0 up, 49 down), 29 (17 up, 12 down), 13 (9 up, 4 down) genes in cerebral cortex, 40 (8 up, 32 down), 11 (7 up, 4 down), 137 (63 up, 74 down) in brainstem, 27 (14 up, 13 down), 33 (18 up, 15 down), 60 (29 up, 31 down) in spinal cord were identified. Among the regulated gene, IL-1β and IL-6 were sustainable enhanced in brain stem, while PKACβ and RGS4 were up-regulated throughout cerebral cortex, brainstem and spinal cord in 3 and 6 month of nerve injury. Intriguingly, subcellular distribution of RGS4 in above three regions was dependent on the functional correlation of PKA and IL-1β.

**Conclusion:**

Data herein indicated that brachial plexus avulsion could efficiently initiate and perpetuate the brain reorganization. Network involved IL-1β and RGS4 signaling might implicate in the re-establish and strengthening of the local circuits at the cortical and subcortical levels.

## Backgrounds

Neuroplasticity is the changing of neurons and the organization of their networks, which may happen through adding new cells or changing of the strength of the connections between neurons. For years it was believed that peripheral injuries could trigger a series of phenotypic changes [1,2], such as neuronal reaction and chromatolysis, even functional plasticity and brain reorganization [3,4]. It was also reported that these alterations of neural substrates occurred with time dependent manner. Namely, rapid changes within minutes are likely due to unmasking of latent synapses, while the changes over a longer time are involve many mechanisms including long-term potentiation, axonal regeneration and sprouting [5,6].

Brachial plexus is formed by the union of the ventral primary rami of the spinal nerve, C5-C8 and T1 [7], which is a complex network of nerves which extends from the neck to the axilla and supplies motor, sensory, and sympathetic fibers to the upper extremity. Accordingly, brachial plexus avulsion usually results in a constant crushing and intermittent shooting pain, even the arm paralysis [8,9]. Recently, brain reorganization was reported to be induced by brachial plexus avulsion.

As well known, cells must integrate the signals that they receive from multiple pathways in order to respond efficiently to environmental cues. But, what happened with regard to brachial plexus avulsion? Up to now, many studies documented that peripheral nerve injury is tightly controlled by cytokines, G-protein coupled receptor pathways [10]. IL-1β and IL-6 in particular, they are not only implicated in the central inflammatory response in glial cells, but also in neuron actions, such as rapid changes in membrane ion currents, activation of neuron-specific CREB and the sphingomyelinase/ceremide pathways [11]. Importantly, these cytokines may eventually evolve into local circuitry and complex loops between cortical and subcortical locations [12].

For G-protein signaling, the emerging picture of G protein signaling (RGS) proteins reveals a highly diverse, multifunctional signaling network, which could permit fine-tuning of its interaction with cytokines [13-15]. This interaction presents the intriguing possibility to regulate signal capacity involving peripheral nerve injury. Accordingly, the present study was determined to examine brachial plexus avulsion induced specific gene expression, as well as the orchestrated G-protein signaling network in the CNS.

## Methods

### Animal preparation

All animal experiments were carried out in accordance with the guidelines and regulations for animal experimentation, NIH and Fudan University. Adult C57BL/6 mice weighing 18-20 g were used in the current experiment (Scientific Animal Center in Shanghai Medical College, Fudan University). The animals were housed in groups (5 per cage) in a controlled environment on a 12 h light-dark cycle, and allowed to acclimate for a minimum of 5 days before conducting experiments. Water and food were available at all times.

Brachial plexus nerve root avulsion was performed based on established methodologies [3]. Briefly, mouse was anesthetized by i.p. injection of sodium pentobarbital (40-50 mg/kg, Shanghai reagent company, Shanghai, China), then was put in prostate position. Incision was made from the occiput to the scapular angulus superior with 4 cm in length. When the muscles were drawn to one side, spinal cord was gently pulled to the left side, the left radix dorsails and radix ventralis from C5 to T1 were exposed and the nerve roots were avulsed from spinal cord. Animal's body temperature was maintained at 37°C throughout the experiment, no post-operation infection occurred. 1, 3 and 6 months later, animals were scarified by decapitation, and brain tissue were removed and stored rapidly.

### BiostarM-140 s microarray

For mRNA isolation, cortex, brainstem and spinal cord were dissected and pooled from five animals in each group, which was to decrease differences attributable to individual variability and increase the statistically power of these experiments. Tissue were mechanically homogenized, mRNA was extracted by UNIzol reagent. After that, mRNA was treated with RNase-free DNase I (Takara, Japan). BiostarM-140 s microarray was performed, which contains probe sets for detection of 14,000 transcripts. The mRNA was labeled in a reverse transcription reaction in the presence of Cy3-dCTP and Cy5-dCTP. The hybridization signals were scanned with ScanArray 4000, each set of gene expression (operation/control) was expressed as ratio of Cy3 to Cy5. Data processing was performed on GenePix Pro 3.0. All arrays were normalized together as one experiment to reduce non-biological variability.

### Real time PCR

Cerebral cortex, brainstem and spinal cord were dissected (50 mg, n = 5), mRNA was extracted by UNIzol reagent and treated with RNase-free DNase I (Takara, Japan). Reverse transcription using random hexamers was performed with Omniscript reverse transcriptase (QIAGEN). Briefly, 20-μl reactions contained DNase-treated RNA, deoxynucleoside triphosphate mix, 1 μM random hexamer primer, 1 U of RNase inhibitor (Ambion), and Omniscript reverse transcriptase. Reactions incubated at 37°C for 1 h, followed by 93°C inactivation for 5 min.

Real time PCR analysis was performed with SYBR Green I (Takara, Japan). Briefly, 50 μl reactions contained cDNA; 0.5 μM of primers specific for IL-1β (sense: 5'-CTCCATGAGCTTTGTACAAGG-3'; antisense: 5'-TGCTGATGTACCAGTTGGGG-3'), IL-6 (sense: 5'-CTCTCCGCAAGAGACTTCCA-3'; antisense: 5'-TGGTCTTCTGGAGTTCCGTT-3'), RGS4 (sense: 5'-CCGGCTTCTTGCTTGAGGAGTG-3'; antisense: 5'-ATCCAGGTTCACATTCATGACT-3'), PKACβ (sense: 5'-AGAAAGCAGGCACTCGTACA-3'; antisense: 5'-AAAGGAGACCGAAAACATGG-3'); 25 μl PCR master mix. PCR was performed in ABI PRISM 7900HI (Applied Biosystems) as follows: 50°C for 2 min and 95°C for 10 min, followed by 40 cycles of 95°C for 15 sec and 60°C for 1 min. Endogenous control (GAPDH) was used for each sample in the same plate, minimizing any effect of plate-to-plate variability. Gene expression was quantified with the 2−ΔΔCt method, which computed the percentage change relative to control.

### ELISA analysis of IL-1β and IL-6 production

IL-1β and IL-6 production in brainstem (50 mg) were assessed by sandwich ELISA according to the manufacturer's instructions (R&D Systems). A 96-well plate was coated with 2 μg/ml monoclonal anti-mouse IL-1β or IL-6 at 4°C overnight and then blocked with 1% BSA in PBS for 1 h. The plates were washed three times with PBS containing 0.2% Tween 20 (PBST). Aliquots of tissue lysate was diluted to 100 μl with HBSS, added to the plates, and incubated for 2 h at room temperature. The plates were washed three times with PBS, 100 μl aliquots of 0.1 μg/ml biotinylated mouse IL-1β or IL-6 affinity-purified polyclonal detection antibody were added and incubated for 2 h. After further three washes with PBST, the immune complexes were colorimetrically detected using HRP-streptavidin conjugate. The reaction was stopped by1 M H_2_SO4. The absorbance at 450 nm in each well was measured by microplate reader (BioRad). Experiments were independently performed three times and the data are represented as the mean ± SEM.

### Preparation of plasma membrane and non-membrane fraction

Cerebral cortex, brainstem and spinal cord from each group of mice, or neurons (1 × 10^6^) undergone respective treatment were homogenized in 3-4 volumes (w/v) of homogenization buffer (0.32 M sucrose, 2 mM Na-EGTA, 1 mM NaN3, 5 pg/ml leupeptin, 5 pg/ml pepstatin A, 200 pg/ml phenylmethylsulfonyl fluoride, and 0.01% (v/v) DFP, pH 7.5) and centrifuged at 900 g for 10 min. The supernatant (S1) was pelleted for 1 h at 30,000 g, then was washed with phosphate/EGTA buffer (10 mM sodium phosphate, 2 mM Na-EGTA, 1 mM NaN3, 0.5 mM DTT, 50 pg/ml phenylmethylsulfonyl fluoride, pH 7.5) and resuspended at a final membrane protein concentration of 3-4 mg/ml, which was used as membrane fraction. S1 was collected and total protein was precipitated by 5% Trichloroacetic acid (TCA), which was used as non-membrane fraction. RGS4 expression in each fraction was analyzed by Western Blot.

### Primary neuron culture

Brainstem neurons were from embryonic day 18 C57BL/6 mice. Fetuses were decapitated and collected under sterile conditions. After removing meninges, neurons were dissociated in 0.05% trypsin at 37°C, then washed in DMEM and gently suspended in neuron-defined serum-free Neurobasal medium supplemented with B27. By flow cytometry, neurons were account for 95% of cultures.

### Detergent-free preparation of lipid rafts

The isolation of lipid rafts in the current study was adapted from Lisanti's lab [16,17]. Neurons were scraped into 2 ml of 500 mM sodium carbonate, PH11.0. Homogenization was carried out sequentially in the following order using a loose-fitting Dounce homogenizer (10 strokes), three 10-sec bursts of a Polytron tissue grinder (Brinkmann Instruments, Inc., Westbury, NY) at setting 6, followed by one 30-sec burst at setting 4 and one 30-sec burst at setting 8 of a sonicator equipped with a micro-probe (Heat systems-Ultrasonics, Inc., Plainview, NY). The homogenate was then adjusted to 45% sucrose by the addition of 2 ml of 90% sucrose prepared in MBS at pH 6.8 and placed at the bottom of an ultracentrifuge tube. The lysate was then overlaid with 4 ml of 35% sucrose and 4 ml of 5% sucrose, both prepared in MBS containing 250 mM sodium carbonate at pH 11. The discontinuous gradient was centrifuged at 39,000 rpm for 16-20 hr in a SW41 rotor. A light-scattering band to the 5-35% and 35-45% sucrose interface was collected and the total proteins were separated and analyzed by Western Blot.

### Western blotting

Proteins were resolved in SDS-PAGE gel, then transferred to a polyvinylidene difluoride membrane (GE Healthcare, Little Chalfont, Buckinghamshire, UK). The membrane was blocked in a blocking solution containing 10% non-fat milk and 1% Tween 20 in Tris-buffered saline, and probed with RGS4 (1:1000), PKACβ (1:1000) respectively. Protein band was detected by alkaline phosphatase conjugated secondary antibody (1:5000) and ECF substrate, and scanned in the Storm 860 Imaging System (GE Healthcare). Band intensities were quantified and analyzed with ImageQuant software (GE Healthcare).

### Statistical analysis

Gene ontology, as well as the information about specific genes of interest was obtained from Pubmed. Intensity ratio of cy3 to cy5 was presented for one gene, that was more than 2.0 or less than 0.5 was considered to show prominent differential expression. Results from ELISA, real time PCR and Western Blot were presented as means ± SEM of three experiments. Statistical significance was determined using one-way ANOVA.

## Results

### Regulated genes in cortex, brainstem and spinal cord

The first step in this study was to screen genes that are regulated by brachial plexus avulsion, the comparison of genes allowed us to narrow down the candidates possibly involved in this process. In cerebral cortex, significant upregulation of 0, 17 and 9 transcripts and downregulation of 49, 12 and 4 transcripts were exhibited after 1, 3 and 6 months of brachial plexus avulsion (Additional file 1). When putting the regulated genes into a functional context, we found that several key genes involved in signal transduction. For example, genes encoding casine kinase, potassium voltage-gated channel and heat shock protein were up-regulated in 3 month. Besides that, clusters of genes involved in cell signaling, cell structure, stress and immune responses, such as genes encoding for protein C receptor, angiotensin, plastaglandin E, microtubule associated protein 1B, tubulin, PKACβ and RGS4 were induced by brachial plexus avulsion.

In brainstem, there were 40 (8 up, 32 down), 11 (7 up, 4 down), 137 (63 up, 74 down) transcripts were significantly regulated in1, 3 and 6 months of brachial plexus avulsion (Additional file 2). PKACβ and RGS4 are among the upregulated genes. Other regulated genes are several involved in immune reaction and signal transduction, including microtubule associated protein 1B, tubulin, and tyrosine ligase, cytochrome P450, thymopoietin, PDZ domain, novel nuclear protein, immunoglobulin family, major histocompatibility complex, angiotensin 2, dual-specificity tyrosine kinae, G-protein coupled receptor 56, FK506 binding protein, N-ras protein, phospholipids D, cytoskeleton associated protein, dynactin, forming binding protein, serine peptidase inhibitor, tenascin, low density lipoprotein receptor, TRIP-Br1, glia maturation factor, peroxisome proliferators activated receptor.

In spinal cord, we found an upregulation of 14, 18 and 29 transcripts and downregulation of 13, 15 and 31 transcripts in 1, 3 and 6 month of brachial plexus avulsion (Additional file 3). Several regulated genes were related to synaptic function, such as synaptotagmin, synaptobrevin and protein tyrosine phosphatase. Some were associated with cell metabolism, such as glutamate-cycteine ligase. PKACβ and RGS4 were the remarkable upregulated genes.

### IL-1β and IL-6 production in the central nervous system by brachial plexus avulsion

**Figure 1:**
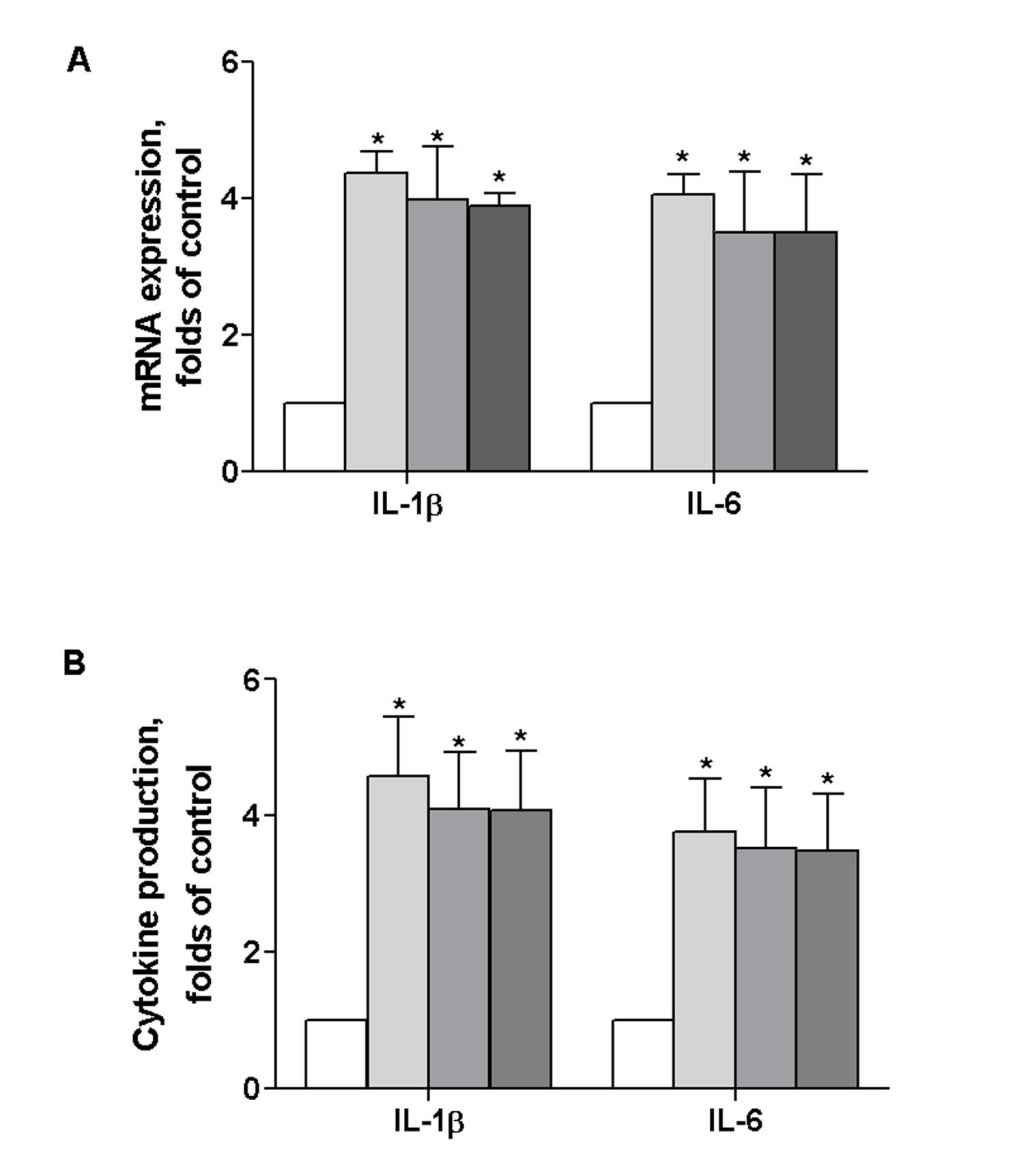
**Kinetic changes in IL-1β and IL-6 expression in brainstem after brachial plexus avulsion**. Mice were divided into 4 groups, control, 1, 3 and 6 month of brachial plexus avulsion (n = 5). IL-1β and IL-6 mRNA expression (A) and protein content (B) in brainstem were evaluated by real time PCR and ELISA assay respectively. The results were normalized against un-operated mouse, data are expressed as mean ± SEM. p < 0.05 *vs control.

Constitutive expression of IL-1β and IL-6 in brain is quite low in basal condition [18]. Based on cDNA microarray data, they could be induced by brachial plexus avulsion with region specific manner. To determine whether this differential regulation occurs only in brainstem, quantification by means of real-time PCR and ELISA assay were performed. We observed a significant up-regulation of IL-1β and IL-6 mRNA expression in brainstem, they were 4.37 ± 0.56 and 4.06 ± 0.49, 3.97 ± 1.38 and 3.51 ± 1.54, 3.89 ± 0.32 and 3.50 ± 1.46 folds of control in 1, 3 and 6 month of operation respectively (Figure 1A). But no changes displayed in cortex and spinal cord (data not shown). IL-1β and IL-6 protein content in brainstem were also elevated (4.57 ± 1.51 and 3.77 ± 1.35, 4.10 ± 1.44 and 3.52 ± 1.54, 4.07 ± 1.51 and 3.49 ± 1.43 folds of control), which clearly matched with their mRNA level (Figure 1B).

### Confirmation of PKACβ and RGS4 gene expression by real-time PCR

**Figure 2:**
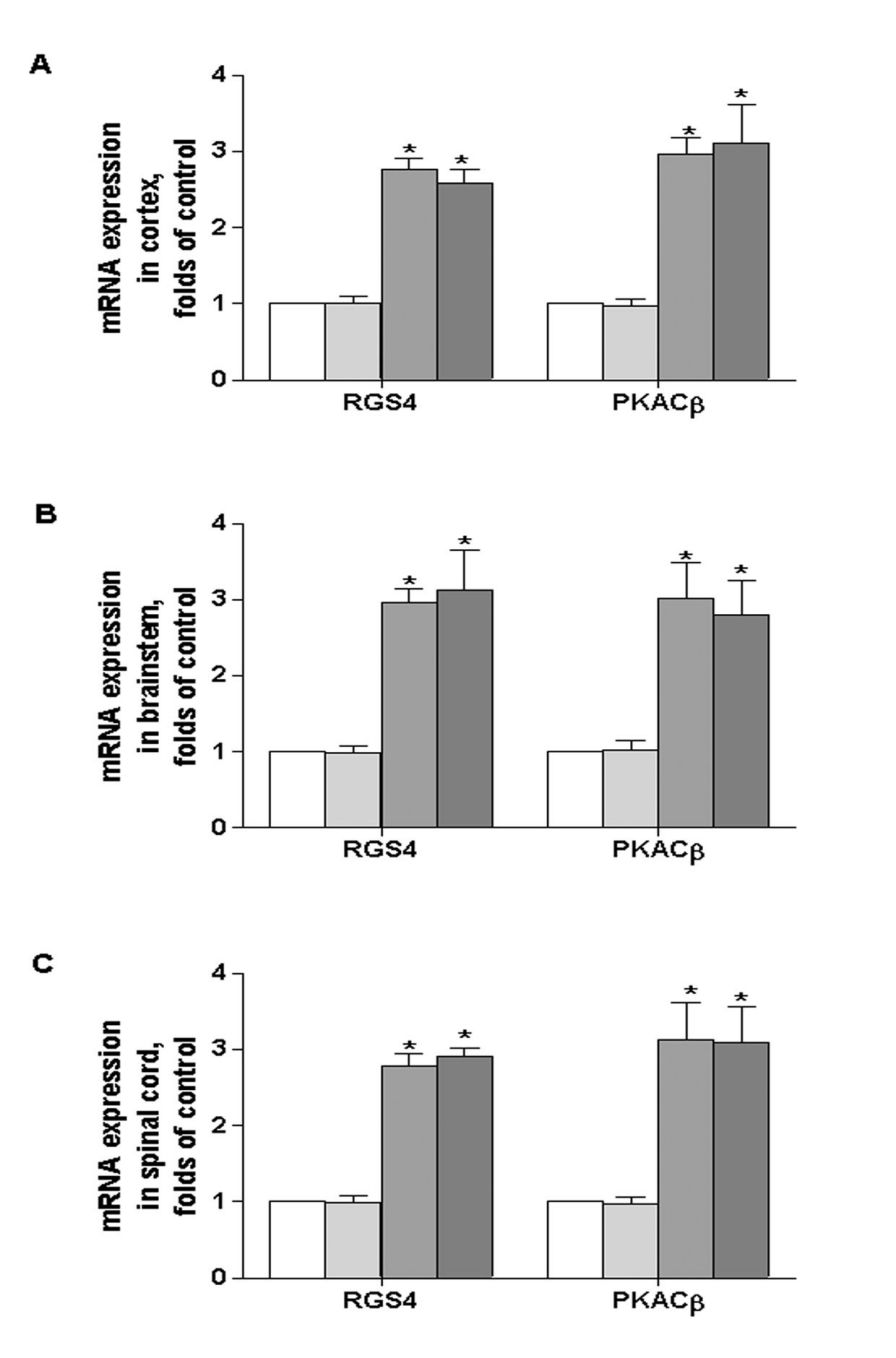
**Kinetic changes in RGS4 and PKACβ expression in brainstem after brachial plexus avulsion**. Mice were divided into 4 groups, control, 1, 3 and 6 month of brachial plexus avulsion (n = 5). RGS4 and PKACβ mRNA expression in cortex (A), brainstem (B) and spinal cord (C) were assayed by real time PCR. The results were normalized against un-operated mouse, data are expressed as mean ± SEM. p < 0.05 *vs control.

The findings by cDNA microarray highlight the complexity in gene expression changes that triggered by brachial plexus avulsion. To validate the genes which are potentially associated with IL-1β signaling, we focused on the genes encoding RGS4 and PKACβ on account of their overall regulation in cortex, brainstem and spinal cord (Table 1). As illustrated in Figure 2, RGS4 and PKACβ mRNA expression in cerebral cortex was 1.02 ± 0.17 and 0.97 ± 0.21, 2.77 ± 0.34 and 2.97 ± 0.48, 2.59 ± 0.39 and 3.12 ± 1.13 folds of control in 1, 3 and 6 month of operation respectively. In brainstem, they were 0.99 ± 0.21 and 1.03 ± 0.28, 2.97 ± 0.39 and 3.02 ± 1.04, 3.12 ± 1.21 and 2.80 ± 1.13 folds of control. In spinal cord, they were 0.99 ± 0.21 and 0.97 ± 0.20, 2.79 ± 0.34 and 3.13 ± 1.10, 2.92 ± 0.21 and 3.09 ± 1.07 folds of control respectively. The results are correlated very well with the data obtained from microarray analysis.

**Table 1:** Genes with confirmed up- or down-regulation

	1 month	3 month	6 month
**Regulated in the motor cortex**			
regulator of G-protein signaling 4 (NM_009062)		↑	↑
protein kinase, cAMP dependent regulatory (NM_008923)		↑	↑
**Regulated in the brain stem**			
IL-1 protein (AJ250429)	↑	↑	↑
interleukin 6 signal transducer (BC058679)	↑	↑	↑
regulator of G-protein signaling 4 (NM_009062)		↑	↑
**Regulated in the spinal cord**			
regulator of G-protein signaling 4 (NM_009062)		↑	↑
protein kinase, cAMP dependent regulatory (NM_008923)		↑	↑

Mice were divided into 4 groups, control, 1, 3 and 6 month of brachial plexus avulsion (n = 5). Cerebral cortex, brainstem and spinal cord were dissected (50 mg, n = 5), mRNA was extracted and Real time PCR analysis was performed with SYBR Green I. Endogenous control (GAPDH) was used for each sample in the same plate, minimizing any effect of plate-to-plate variability. Gene expression was quantified with the 2−ΔΔCt method, which computed the percentage change relative to control.

### Modulation of RGS4 membrane distribution by brachial plexus avulsion

**Figure 3:**
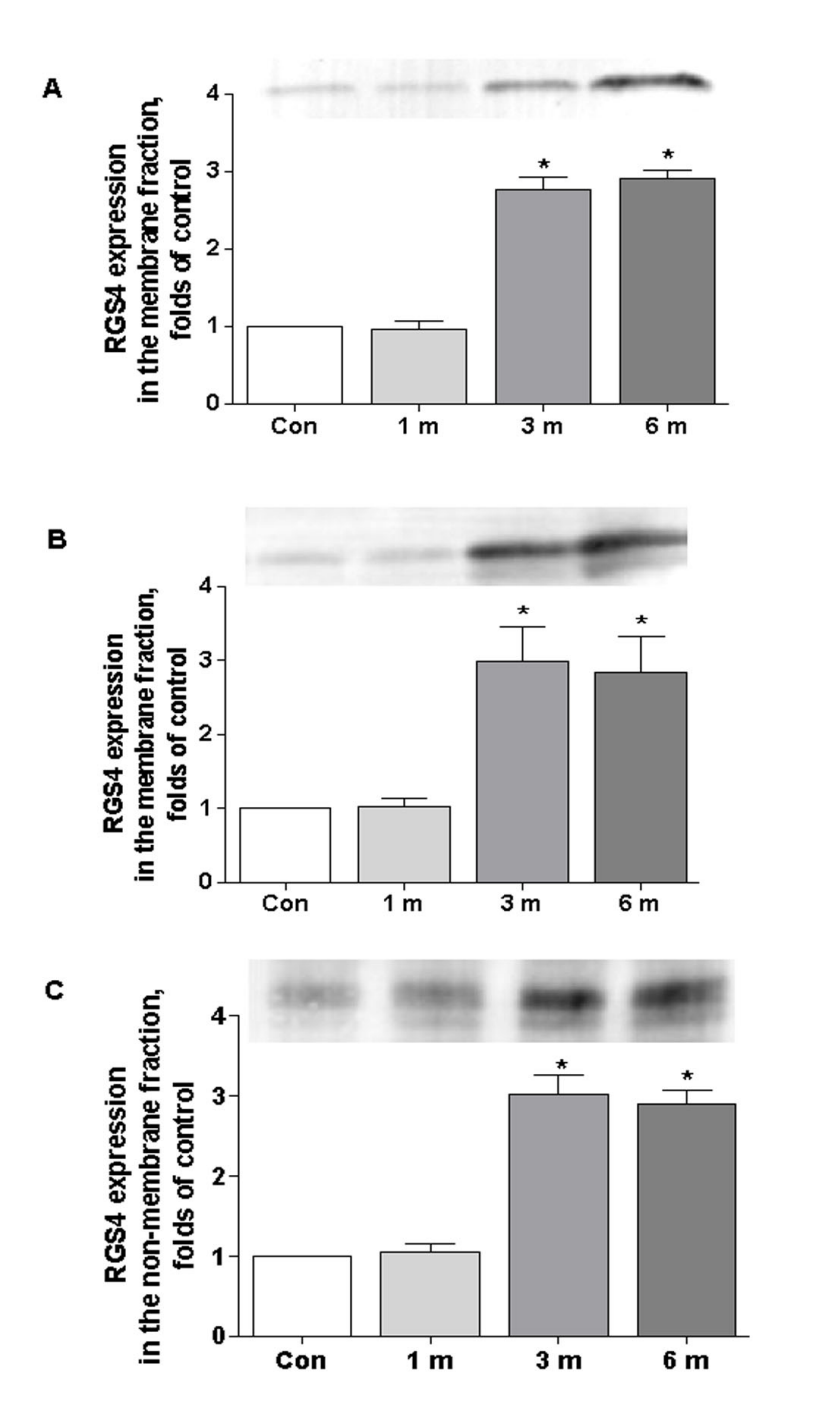
**Modulation of RGS4 membrane distribution by brachial plexus avulsion**. Mice were divided into 4 groups, control, 1, 3 and 6 month of brachial plexus avulsion (n = 5). Western blot analysis of RGS4 in the membrane fraction collected from cortex (A) and spinal cord (B), in the non-membrane fraction in brainstem (C). RGS4 protein was densitometric analyzed and normalized against un-operated mouse, data are expressed as mean ± SEM. p < 0.05 *vs control.

Since the regulation of RGS4 was dependent on its phosphorylation and subcelllular distribution [19], we determined to examine RGS4 expression profiles when challenged with brachial plexus avulsion. As shown in Figure 3, membrane distribution of RGS4 in cerebral cortex and spinal cord began to increase in 3 and 6 month of nerve injury. The relative densities to control were 0.97 ± 0.23, 2.77 ± 0.35, 2.92 ± 0.21 folds of control in cortex, 1.03 ± 0.22, 2.98 ± 1.08, 2.83 ± 1.09 folds of control in spinal cord in 1, 3 and 6 month of nerve injury respectively. However, in brainstem, elevated RGS4 was mainly concentrated in non-membrane fraction, the relative densities to control were 1.05 ± 0.23, 3.03 ± 0.52, 2.90 ± 0.38 folds of control in the three time points.

### Modulation of RGS4 membrane distribution in cultured neuron

**Figure 4:**
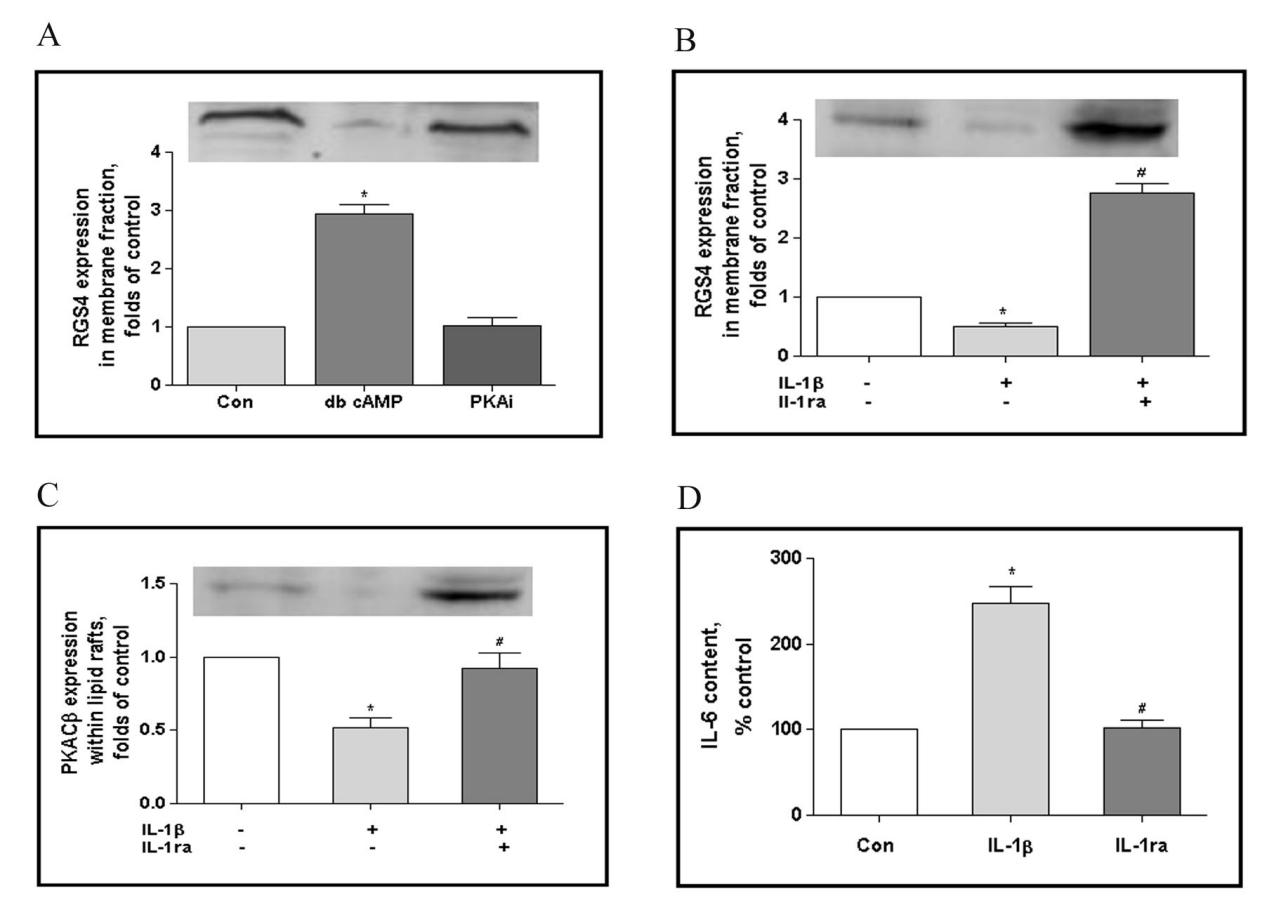
**Modulation of RGS4 membrane distribution in cultured neuron**. Cortical neurons were grown for 10 days, then RGS4 subcellular distribution in the presence or absence of db cAMP, KT5720 (A); IL-1β, IL-1ra (B) was assayed by Western blotting. The localization of PKCβ within lipid rafts microdomain was assayed by discontinuous sucrose centrifugation and Western blotting (C). IL-6 content in the presence or absence of IL-1β, IL-1ra was evaluated by ELISA assay (D). The results were normalized against vehicle treated neurons. Statistical differences were evaluated by ANOVA analysis.*p < 0.05 *vs *control.

Whether IL-1β or PKACβ controlled RGS4 subcellular distribution? As displayed in Figure 4, RGS4 was localized within non-membrane fraction in cultured neurons in basal condition. Administration of cAMP analogue dibutyryl-cAMP (dbcAMP, 0.25 mM, 24 h) resulted in RGS4 translocated into membrane fraction, the relative density to vehicle treatment was 2.93 ± 0.37 folds of control. In contrast, PKA inhibitor, KT5720 (9-n-hexyl derivative of K-252a, 10 μM, 30 min) exposure attenuated RGS4 membrane distribution, the relative density was 1.02 ± 0.30 folds of control. Similar observation was also obtained on the neurons collected from cortex and spinal cord (data not shown).

However, what the mechanism underlying the role of IL-1β on the RGS4 membrane distribution? Figure 4 illustrated that relative density of RGS4 in membrane fraction was attenuated when exposed to IL-1β (20 ng/ml, 24 h) (0.51 ± 0.11 folds of control). IL-1 receptor antagonist (IL-1ra, 10 ng/ml, 24 h) showed significant inversely effect, the relative density of RGS4 was elevated to 2.76 ± 0.38 folds of control. Discontinuous sucrose density centrifugation is designed to specifically quantify the PKACβ distribution within lipid rafts. Notably, IL-1β treatment leads to the decreased PKACβ expression in lipid raft microdomain, relative density was 0.52 ± 0.14 folds of % control. IL-1ra could reverse the effect of IL-1β, PKACβ lipid rafts distribution was restored (0.92 ± 0.24 folds of control). Also, IL-6 content in neuron is efficiently controlled by IL-1β.

## Discussion

Previous study have implicated that peripheral nerve injury induced reorganization is not equally distributed across the neuroaxis. The acute effects of nerve injury appear to be much more pronounced in the brainstem than in cortex [20], while the more protracted phase of effects appears to result from differing extents of secondary functional changes, as well as the more dynamic interactive processes in cortex [21,22]. Thus, it will be interesting to validate the putative targets, by which fine-tuned a spatio-temporal reorganization following brachial plexus avulsion.

In this study, application of microarray analyses, we found that 49 (0 up, 49 down), 29 (17 up, 12 down), 13 (9 up, 4 down) genes in cortex were changed in 1,3 and 6 months of brachial plexus avulsion. In brainstem, 40 (8 up, 32 down), 11 (7 up, 4 down), 137 (63 up, 74 down) genes were identified. Some of these genes are involved in axonal transport, which would be expected to closely link to the actin projection cut down [23,24]. Moreover, the alteration pointed to the hypothesis that the injured environment was dominated by deafferentation induced responses, neural tissue was surrounded by stimulated fibers and reorganized vasculature [25]. In spinal cord, 27 (14 up, 13 down), 33 (18 up, 15 down), 60 (29 up, 31 down) genes were identified. Upregulation of synaptotagmin, synaptobrevin and protein tyrosine phosphatase signified the increased synaptic efficacy and formation of new synaptic buttons of the existing projections [26,27], which might pinpoint the compensatory mechanisms.

Notably, we found that the changes occur after brachial plexus avulsion is relatively specific in the brainstem, IL-1β and IL-6 displayed differential expression profile in brainstem compared with cortex and spinal cord. Based on the report, IL-1β and IL-6 are associated with many brain functions [18]. For example, they have been implicated in the excessive production and processing of β-amyloid precursor protein and the plaque-associated proteins [28]; They can induce the production of various growth and trophic factors, including fibroblast growth factor-2 (FGF-2) [29], transforming growth factor-β (TGF-β) [30], and nerve growth factor (NGF) [31]; They can also stimulate inflammatory mediators, such as phospholipase A2, cyclooxygenase-2 (Cox-2), prostaglandins, nitric oxide, matrix metalloproteinases, collagenase [18], adhesion molecule and other cytokines [32,33]. Combined with our present data, it is reasonable to propose that the orchestrated IL-1β and IL-6 expression induced by brachial plexus avulsion are likely responsible for setting up the cytokines networks.

It is even more striking that RGS4 and PKACβ expression were up-regulated in 3 and 6 month of brachial plexus avulsion, the elevation occurred overall in cortex, brainstem and spinal cord. As well known, heterotrimeric G proteins transduce signals from a wide range of hormone and neurotransmitter receptors at the cell surface to the intracellular environment. Following activation, G proteins interact with well-defined effectors such as phopholipase C, adenylyl cyclase, a number of ion channels and RGS proteins [34,35]. RGS proteins is defined by a conserved 130 amino acid RGS domain, which serve as a GTPase activating protein (GAP) by binding to activated Gα subunits [36,37]. Now, RGS superfamily has more than 30 distinct mammalian proteins, RGS4 has densely labeling in cortex, thalamus and striatum [19]. Kinase activity could affect RGS proteins stability, their interaction with Gα subunits, or their cellular trafficking [38,39]. It has also revealed that RGS phosphorylation appears to be related to its subpopulation of intracellular vesicles [40]. For example, when RGS2 was phosphorylated by PKC, GAP activity was reduced, then Gq/11 signals were enhanced [41]; RGS16 phosphorylation could also reduce its GAP activity toward GRi/o and result in increased adrenergic receptor signals [42], then the paralleled increase in RGS4 and PKA induced by brachial plexus avulsion will be expected to unmask their full functional potential, the orchestrated and dynamical RGS4 subcellular distribution and modulation will be the intriguing intracellular signal in response to brachial plexus avulsion. Our findings support this hypothesis: RGS4 was mainly expressed in membrane fraction in cortex and spinal cord, in non-membrane fraction in brainstem. Since PKACβ expression was along with RGS4, it is speculated that RGS4 differential expression profile may be due to their relevance. As expected, direct analyzing using cultured neuron displayed that RGS4 membrane distribution was dependent on PKA activation.

Several studies have documented that cAMP-CREB pathway is highly involved in brain inflammatory processes, whose cellular responses to neurotransmitters, synaptic plasticity, differentiating factors and stressors were mainly concentrated in lipid rafts [43-45]. IL-1β-IL-6 signaling in neuron are known to be the upstream of cAMP and CREB [46,47]. In the present study, IL-1β could disrupt RGS4 membrane distribution by promoting PKA shuttle out of lipid rafts. We therefore presumed that the alteration in RGS4 membrane turnover in brainstem was derived from the presence of IL-1β. There might exist alternative RGS4 related signaling pathway in cortex, spinal cord and brainstem.

Altogether, these data support the ability of brachial plexus avulsion on the brain reorganization. After nerve injury, coordinated tissue remodeling and amplified the mounting cellular response in cortex, brainstem and spinal cord was efficiently initiated and perpetuated. IL-1β-IL-6 signaling in brainstem in the early stage (1 month) appears to enhance the perceptions of compensatory modalities, and may serve to reinforcement of feedback activity in response to nerve injury. In the later stage (3 and 6 month), it may triggered differed G-protein signal events within CNS, which might consequently function to re-establish or strengthening of the local circuits at cortical and subcortical levels.

## Conclusion

The present study showed that brachial plexus avulsion lead to both specific as well as more global changes in gene expression in cortex, brainstem and spinal cord. The regulated genes at acute or longer times display interesting similarities or differences among the three brain regions, which may contribute to the different aspects of brain responses to brachial plexus avulsion. For example, IL-1β and IL-6 expression were upregulated only in brainstem, while RGS4 and PKACβ expression could be induced overall in cortex, brainstem and spinal cord. Importantly, RGS4 displayed differential distribution, namely, in membrane fraction in cortex and spinal cord, while in non-membrane fraction in brainstem. This subcellular distribution was dependent on the functional correlation between IL-1β and PKACβ. Thereby, we assumed that temporal and spatial IL-1β-IL-6 signaling in brain might function to re-establish or strengthening of the local circuits at cortical and subcortical levels.

## Abbreviations

cAMP: cyclic AMP; CNS: central nervous system; CREB: cAMP response element-binding protein; GAP: GTPase activating protein; IL-1β: interleukin 1-β; IL-6: interleukin-6; PKACβ: protein kinase A catalytic subunit β; RGS4: regulator of G protein signaling 4.

## Competing interests

The authors declare that they have no competing interests.

## Authors' contributions

HZ did design, data acquisition, analysis, and writing. YG and JL revised the manuscript. PL did animal experiment. All approved the final version.

## Supplementary Material

Additional file 1 **Functional classification of the annotated genes that show differentiated expressions in the motor cortex following brachial plexus axotomy**. Intensity ratio of cy3 to cy5 was presented for one gene, that was more than 2.0 or less than 0.5 was considered to show prominent up- or down-regulated expression. Click here for file

Additional file 2 **Functional classification of the annotated genes that show differentiated expressions in the brain stem following brachial plexus axotomy**. Intensity ratio of cy3 to cy5 was presented for one gene, that was more than 2.0 or less than 0.5 was considered to show prominent up- or down-regulated expression. Click here for file

Additional file 3 **Functional classification of the annotated genes that show differentiated expressions in the spinal cord following brachial plexus axotomy**. Intensity ratio of cy3 to cy5 was presented for one gene, that was more than 2.0 or less than 0.5 was considered to show prominent up- or down-regulated expression. Click here for file
