# Supplementary material for: Functional cooperation of of IL-1β and RGS4 in the brachial plexus avulsion mediated brain reorganization
Source: J Brachial Plex Peripher Nerve Inj. 2010 Dec 7;5:18. doi: 10.1186/1749-7221-5-18 (PMC3017042; doi:10.1186/1749-7221-5-18)
Supplement: Additional file 1 — Functional classification of the annotated genes that show differentiated expressions in the motor cortex following brachial plexus axotomy. Intensity ratio of cy3 to cy5 was presented for one gene, that was more than 2.0 or less than 0.5 was considered to show prominent up- or down-regulated expression. [file 1749-7221-5-18-S1.DOC]

**Additional file 1**

|  | ***1 month*** | ***3 month*** | ***6 month*** |
| --- | --- | --- | --- |
| **Transcription factors and DNA binding protein** |  |  |  |
| DNA topoisomerase II (MUSTOP2) | 0.265 |  |  |
| pleckstrin homology domain interacting protein (XM_358384) | 0.332 |  |  |
| zinc finger protein 82 (BC025629) | 0.39 |  |  |
| zinc finger protein 36 (NM_001001806) |  | 0.47 |  |
| novel nuclear protein 1 (BC065993) |  | 3.483 | 3.399 |
| dicer-like protein (AF430845) |  |  | 2.128 |
| **Immune factors** |  |  |  |
| macrophage receptor with collagenous structure (NM_010766) | 0.303 |  |  |
| monocyte to macrophage differentiation-associated 2 (NM_175217) | 0.421 |  |  |
| protein C receptor (BC028755) | 0.469 | 0.331 |  |
| oxidative stress induced growth inhibitor 1 (BC006032) |  | 2.093 |  |
| suppressor of cytokine signaling 5 (BC053015) |  |  | 2.145 |
| **Hormone and transmitter system** |  |  |  |
| methionine adenosyltransferase II (BC058360) | 0.364 | 0.405 | 0.399 |
| angiopoietin-like 4 (BC021343) | 0.426 | 0.414 |  |
| prostaglandin E receptor 4 (BC011193) | 0.44 | 0.377 |  |
| **Signal transduction** |  |  |  |
| cationic amino acid transporter (MMU70859) | 0.224 |  |  |
| mitogen-activated protein kinase 8 interacting protein 2 (NM_021921) | 0.259 |  |  |
| calcium-activated potassium channel (MMU09383) | 0.266 |  |  |
| tyrosine phosphatase LAR (AF300943) | 0.271 |  |  |
| ADP-ribosylation factor 3 (NM_007478) | 0.297 |  |  |
| mitogen activated protein kinase 13 (BC001992) | 0.362 |  |  |
| transmembrane protein 138 (BC058237) | 0.367 |  |  |
| developmentally regulated GTP binding protein 2 (BC082564) | 0.381 |  |  |
| G protein-coupled receptor 56 (NM_018882) | 0.408 |  |  |
| G protein-coupled receptor associated sorting protein 1 (NM_026081) | 0.44 |  |  |
| serine/threonine kinase 16 (NM_011494) | 0.447 |  |  |
| ubiquitin-conjugating enzyme E2N (BC067069) | 0.47 |  |  |
| sodium channel 21 (MUSSC21R) |  | 0.24 |  |
| putative seven transmembrane spanning receptor (AJ300198) |  | 0.312 |  |
| AN1, ubiquitin-like, homolog (XM_132758) |  | 0.452 |  |
| dual specificity phosphatase 18 (NM_173745) |  | 2.225 |  |
| regulator of G-protein signaling 4 (NM_009062) |  | 2.332 | 3.33 |
| transmembrane protein 53 (BC039805) |  | 2.369 |  |
| protein kinase, cAMP dependent regulatory (NM_008923) |  | 3.483 | 3.399 |
| casein kinase 1 (BC063083) |  | 4.123 |  |
| potassium voltage-gated channel (NM_001003824) |  | 5.369 |  |
| heat shock protein 8 (NM_031165) |  | 5.936 |  |
| protein kinase, AMP-activated (NM_001013367) |  | 7.036 |  |
| AP2 associated kinase 1 (NM_177762) |  |  | 0.25 |
| AXL receptor tyrosine kinase (BC058230) |  |  | 2.277 |
| dual-specificity tyrosine-phosphorylation regulated kinase 1a (BC034550) |  |  | 2.752 |
| **Synapse** |  |  |  |
| putative synaptopodin (MMU278123) | 0.221 |  |  |
| claudin 15 (BC023428) | 0.382 |  |  |
| **Cytoskeleton and motility proteins** |  |  |  |
| microtubule-associated protein 1B (MMMAP1B) | 0.266 | 0.356 |  |
| dynactin 1 (BC066061) | 0.386 |  |  |
| myotubularin related protein 1 (BC056376) | 0.42 |  |  |
| Tubulin (BC094022) | 0.471 | 0.418 |  |
| **Miscellaneous** |  |  |  |
| mitochondrial ribosomal protein L4 (BC023078) | 0.139 |  |  |
| putative phosphoinositide 5-phosphatase type II (MMU96724) | 0.228 |  |  |
| sulfotransferase family 1A (BC005413) | 0.303 |  |  |
| ATPase (BC015262) | 0.317 |  |  |
| CDP-diacylglycerol synthase 1 (NM_173370) | 0.324 |  |  |
| L-2-hydroxyglutarate dehydrogenase (BC016226) | 0.33 |  |  |
| monoglyceride lipase (NM_011844) | 0.331 |  |  |
| mannose-6-phosphate receptor (NM_010749) | 0.342 |  |  |
| apolipoprotein F (NM_133997) | 0.363 |  |  |
| phosphatidylinositol glycan anchor biosynthesis (BC058979) | 0.382 |  |  |
| tumor protein p53 inducible nuclear protein 2 (BC043086) | 0.388 |  |  |
| NADPH dependent diflavin oxidoreductase 1 (BC049789) | 0.389 |  |  |
| ganglioside-induced differentiation-associated protein 1-like 1 (BC019941) | 0.397 |  |  |
| Aprataxin (BC068309) | 0.41 |  |  |
| branched chain aminotransferase 2 (BC048072) | 0.412 |  |  |
| ATP-binding cassette (NM_013855) | 0.415 |  |  |
| BCL2-associated transcription factor 1 (BC086624) | 0.424 |  |  |
| protein-L-isoaspartate (D-aspartate) O-methyltransferase 1 (BC040750) | 0.441 |  |  |
| ATP synthase (BC048777) | 0.456 |  |  |
| cysteine dioxygenase 1 (BC020375) | 0.483 |  |  |
| cytochrome P450, family 2 (NM_010000) | 0.488 |  |  |
| serine (or cysteine) peptidase inhibitor (NM_011458) | 0.498 |  |  |
| apolipoprotein L (XM_484487) |  | 0.496 |  |
| death associated protein kinase (X97048) |  | 0.497 |  |
| NADH dehydrogenase (BC006660) |  | 2.036 |  |
| electron transferring flavoprotein (NM_145615) |  | 2.04 |  |
| 5-methyltetrahydrofolate-homocysteine methyltransferase (XM_138431) |  | 2.051 |  |
| tetraspanin 5 (BC058695) |  | 2.095 |  |
| mitochondrial fission regulator 1 (BC042471) |  | 2.103 |  |
| sestrin 1 (BC055753) |  | 2.182 |  |
| tryptophan 2,3-dioxygenase (NM_019911) |  | 2.415 |  |
| MYST histone acetyltransferase 2 (BC057102) |  |  | 0.39 |
| glia maturation factor-beta (AF297220) |  |  | 0.495 |
| cyclin A1 (X84311) |  |  | 2.083 |
| glutathione S-transferase (NM_026764) |  |  | 8.27 |
