# Supplementary material for: Functional cooperation of of IL-1β and RGS4 in the brachial plexus avulsion mediated brain reorganization
Source: J Brachial Plex Peripher Nerve Inj. 2010 Dec 7;5:18. doi: 10.1186/1749-7221-5-18 (PMC3017042; doi:10.1186/1749-7221-5-18)
Supplement: Additional file 2 — Functional classification of the annotated genes that show differentiated expressions in the brain stem following brachial plexus axotomy. Intensity ratio of cy3 to cy5 was presented for one gene, that was more than 2.0 or less than 0.5 was considered to show prominent up- or down-regulated expression. [file 1749-7221-5-18-S2.DOC]

**Additional file 2**

|  | ***1 month*** | ***3 month*** | ***6 month*** |
| --- | --- | --- | --- |
| **Transcription factors and DNA binding protein** |  |  |  |
| pleckstrin homology domain interacting protein (XM_358384) | 0.338 |  |  |
| TBX2 protein (AF172088) | 0.451 |  |  |
| zinc finger protein 148 (NM_011749) |  | 0.301 |  |
| pleckstrin homology domain-containing, family A (NM_031256) |  |  | 0.198 |
| nuclear protein (D83033) |  |  | 0.408 |
| SH3-binding kinase 1 (BC031759) |  |  | 2.114 |
| ribonuclease III (BC088999) |  |  | 2.172 |
| centrosomal protein 70 (BC050819) |  |  | 2.532 |
| zinc finger protein 664 (XM_355639) |  |  | 2.627 |
| PDZ domain containing 8 (XM_140761) |  |  | 3.079 |
| novel nuclear protein 1 (BC065993) |  |  | 3.275 |
| **Immune factors** |  |  |  |
| interleukin 6 (BC058679) | 2.562 | 2.221 | 3.502 |
| IL-1 protein (AJ250429) | 2.686 | 2.225 | 3.211 |
| murinoglobulin 1 (BC051037) |  |  | 0.198 |
| insulin-like growth factor I receptor (NM_010513) |  |  | 0.202 |
| lymphocyte antigen 6 complex (NM_148939) |  |  | 0.271 |
| interferon (alpha and beta) receptor 2 (BC071225) |  |  | 0.292 |
| latent transforming growth factor beta binding protein 4 (NM_175641) |  |  | 0.309 |
| chemokine (C-C motif) receptor-like 2 (NM_017466) |  |  | 0.342 |
| IK cytokine (BC014739) |  |  | 0.453 |
| MHC class I related protein 1 (AF010451) |  |  | 0.473 |
| growth hormone receptor (BC075720) |  |  | 0.497 |
| T cell-specific serine protease (MUSSPTCS) |  |  | 2.011 |
| transforming growth factor (BC063260) |  |  | 2.25 |
| oxidative stress induced growth inhibitor 1 (BC006032) |  |  | 2.425 |
| HLA-B-associated transcript 3 (NM_057171) |  |  | 2.477 |
| CD300e antigen (BC039971) |  |  | 2.712 |
| immunoglobulin superfamily (NM_177591) |  |  | 5.907 |
| major histocompatibility complex class Ib (AY211082) |  |  | 9.944 |
| **Hormone and transmitter system** |  |  |  |
| methionine adenosyltransferase II (BC058360) | 0.403 |  |  |
| adrenergic receptor kinase (BC033272) | 0.451 |  |  |
| insulin degrading enzyme (BC041675) | 2.051 |  |  |
| thymopoietin zeta (MMU39073) | 3.231 |  |  |
| angiopoietin 1 (BC067410) |  |  | 0.164 |
| glucocorticoid receptor (X04435) |  |  | 0.456 |
| opioid binding protein/cell adhesion molecule-like (NM_177906) |  |  | 0.472 |
| GABA-B1a receptor (AF114168) |  |  | 2.723 |
| glutamate receptor (NM_023168) |  |  | 2.727 |
| angiopoietin 2 (NM_007426) |  |  | 3.16 |
| **Signal transduction** |  |  |  |
| cationic amino acid transporter (MMU70859) | 0.282 |  |  |
| dual specificity phosphatase 27 (NM_001033344) | 0.331 |  |  |
| MAP3K12 binding inhibitory protein 1 (BC002277) | 0.416 |  |  |
| catenin (cadherin associated protein) (BC046589) | 0.417 |  |  |
| protein kinase, cAMP dependent regulatory (NM_008923) |  | 4.315 | 4.429 |
| protein kinase inhibitor (BC048244) | 0.438 |  |  |
| G protein-coupled receptor 56 (NM_018882) | 0.457 |  |  |
| protein phosphatase 2 (BC065100) | 0.459 |  |  |
| transmembrane protein 106A (BC022145) | 2.159 |  |  |
| regulator of G-protein signaling 4 (NM_009062) |  | 3.022 | 3.102 |
| transmembrane protein 53 (BC039805) |  | 3.917 | 2.36 |
| P21 activated kinase-3 (MMU39738) |  |  | 0.065 |
| adenylyl cyclase (MMU30602) |  |  | 0.077 |
| protein tyrosine phosphatase (MMU84411) |  |  | 0.093 |
| Optineurin (BC072590) |  |  | 0.109 |
| receptor protein tyrosine phosphatase (AF244125) |  |  | 0.118 |
| plasma membrane Ca++ transporting ATPase (AY560895) |  |  | 0.119 |
| chloride channel CLIC-like 1 (BC003247) |  |  | 0.128 |
| A kinase anchor protein 14 (NM_001033785) |  |  | 0.187 |
| nuclear transport factor 2 (NM_026532) |  |  | 0.207 |
| tyrosine kinase (BC052421) |  |  | 0.235 |
| ubiquitin-conjugating enzyme E2N (BC067069) |  |  | 0.256 |
| serine proteinase inhibitor 2A (AY862185) |  |  | 0.318 |
| adenylate kinase 2 (NM_016895) |  |  | 0.372 |
| AXL receptor tyrosine kinase (BC058230) |  |  | 0.377 |
| Clathrin (BC079897) |  |  | 0.4 |
| PFTAIRE protein kinase 1 (BC068134) |  |  | 0.409 |
| arrestin domain containing 1 (BC004091) |  |  | 0.411 |
| calmodulin 3 (BC050926) |  |  | 0.428 |
| Calnexin (NM_025869) |  |  | 0.459 |
| nuclear pore membrane glycoprotein (AF516680) |  |  | 0.47 |
| potassium voltage-gated channel (BC004629) |  |  | 0.481 |
| calsyntenin 3 (NM_153508) |  |  | 2.085 |
| adaptor protein complex AP-1 (BC052692) |  |  | 2.099 |
| vesicle-associated membrane protein 2 (BC055105) |  |  | 2.119 |
| multiple substrate lipid kinase (NM_023538) |  |  | 2.274 |
| G protein pathway suppressor 1 (BC003350) |  |  | 2.309 |
| vezatin, adherens junctions transmembrane protein (NM_172538) |  |  | 2.389 |
| heat shock protein 8 (NM_031165) |  | 2.735 | 2.55 |
| casein kinase 1 (BC063083) |  |  | 2.585 |
| Rab3D (AF263365) |  |  | 2.705 |
| protein-tyrosine-phosphatase (D64141) |  |  | 2.871 |
| dual-specificity tyrosine-(Y)-phosphorylation regulated kinase 1a(BC034550) |  |  | 3.136 |
| G protein-coupled receptor 56 (NM_018882) |  |  | 3.335 |
| mitogen-activated protein kinase 8 interacting protein 2 (NM_021921 |  |  | 3.349 |
| FK506 binding protein 8 (BC027808) |  |  | 3.454 |
| N-ras protein (X13664) |  |  | 3.564 |
| phospholipase D family (BC076586) |  |  | 3.658 |
| **Synapse** |  |  |  |
| endophilin II (MMU58885) | 0.295 |  |  |
| synaptobrevin like 1 (NM_011515) |  |  | 0.177 |
| Eph receptor A4 (BC052164) |  |  | 0.355 |
| syntaxin 3 (BC056949) |  |  | 0.394 |
| synaptophysin-like protein (BC048846) |  |  | 0.478 |
| synaptotagmin 1 (BC048187) |  |  | 2.504 |
| **Cytoskeleton and motility proteins** |  |  |  |
| Tubulin (NM_011655) | 0.145 | 0.111 | 0.441 |
| microtubule-associated protein 1B (MMMAP1B) | 0.216 | 0.216 | 0.125 |
| tubulin tyrosine ligase-like family (NM_028921) |  |  | 0.19 |
| myosin VC (XM_198225) |  |  | 0.21 |
| Integrin (NM_001001309) |  |  | 0.253 |
| phosphatase and actin regulator 1 (NM_198419) |  |  | 0.444 |
| myotubularin related protein 1 (BC056376) |  |  | 0.45 |
| sorting nexin associated golgi protein 1 (BC063089) |  |  | 0.463 |
| echinoderm microtubule associated protein like 1 (BC053094) |  |  | 2.094 |
| myelin transcription factor 1 (NM_008665) |  |  | 2.371 |
| microfibrillar-associated protein 3 (BC058567) |  |  | 2.479 |
| cytoskeleton-associated protein 4 (NM_175451) |  |  | 3.147 |
| dynactin 1 (BC066061) |  |  | 3.995 |
| formin binding protein 11 (AF135439) |  |  | 8.939 |
| **Miscellaneous** |  |  |  |
| neurogenic differentiation 6 (BC087831) | 0.139 |  |  |
| UDP-glucuronosyltransferase (MMUDPGT) | 0.178 |  |  |
| putative emu1 protein (MMU416093) | 0.211 |  |  |
| aspartyl beta-hydroxylase (AF289487) | 0.221 |  |  |
| mitochondrial ribosome recycling factor (BC019787) | 0.234 |  |  |
| putative phosphoinositide 5-phosphatase type II (MMU96724) | 0.299 |  |  |
| glutamate-cysteine ligase (BC019374) | 0.309 |  |  |
| Aprataxin (BC068309) | 0.345 |  |  |
| ATP synthase (BC048777) | 0.364 |  |  |
| oxoglutarate dehydrogenase (BC049104) | 0.385 |  |  |
| TBC1 domain family (NM_173186) | 0.386 |  |  |
| N-acetyltransferase 2 (NM_010874) | 0.402 |  |  |
| cysteine dioxygenase 1 (BC020375) | 0.44 |  |  |
| ATP-binding cassette (BC020388) | 0.447 |  |  |
| folate hydrolase (NM_016770) | 0.448 |  |  |
| apolipoprotein F (NM_133997) | 0.451 |  |  |
| cytochrome P450 (BC011222) | 0.473 |  | 0.275 |
| mannose-6-phosphate receptor (NM_010749) | 0.478 |  |  |
| pancortin-3 (D78264) | 2.102 |  |  |
| Bcl6 interacting corepressor (BC058656) | 2.171 |  |  |
| acyl-CoA thioesterase 1 (BC040749) | 2.413 |  |  |
| monoacylglycerol O-acyltransferase 2 (NM_177448) |  | 0.328 |  |
| soluble adenylyl cyclase (NM_173029) |  | 2.091 |  |
| glycine C-acetyltransferase (NM_013847) |  |  | 0.061 |
| pyruvate dehydrogenase (MUSPDHA2A) |  |  | 0.089 |
| cyclin C (MMU62638) |  |  | 0.098 |
| cyclophilin-related protein (MUSCYCLORA) |  |  | 0.101 |
| betaine-homocysteine methyltransferase 2 (BC013515) |  |  | 0.11 |
| sortilin 1 (BC056343) |  |  | 0.121 |
| UDP galactosyltransferase 8A (NM_011674) |  |  | 0.132 |
| cell division cycle associated 2 (BC067009) |  |  | 0.161 |
| glutamine fructose-6-phosphate transaminase 1 (NM_013528) |  |  | 0.18 |
| axin 1 (NM_009733) |  |  | 0.183 |
| aspartyl beta-hydroxylase (AF289487) |  |  | 0.213 |
| phosphatidylinositol-4-phosphate 5-kinase, type II (NM_054097) |  |  | 0.218 |
| pantothenate kinase 2 (NM_153501) |  |  | 0.28 |
| lysozyme-like 6 (BC048617) |  |  | 0.329 |
| Bcl6 interacting corepressor (BC058656) |  |  | 0.33 |
| monoacylglycerol O-acyltransferase 2 (NM_177448) |  |  | 0.338 |
| glycine decarboxylase (BC017135) |  |  | 0.342 |
| caspase-7 (Y13088) |  |  | 0.348 |
| mitochondrial ribosomal protein L47 (BC029173) |  |  | 0.351 |
| mannoside acetylglucosaminyltransferase 3 (BC053040) |  |  | 0.371 |
| Proteasome (NM_011184) |  |  | 0.416 |
| Regucalcin (BC012710) |  |  | 0.431 |
| formiminotransferase cyclodeaminase (BC024078) |  |  | 0.457 |
| branched chain aminotransferase 2 (BC048072) |  |  | 0.476 |
| N-acetylglucosamine-6-O-sulfotransferase (AB040710) |  |  | 0.483 |
| phosphatidylserine decarboxylase (BC070408) |  |  | 0.489 |
| tumor protein D53 (AF004428) |  |  | 0.49 |
| NAD(P)H dehydrogenase (NM_008706) |  |  | 2.027 |
| farnesyl diphosphate farnesyl transferase 1 (BC054722) |  |  | 2.057 |
| 4-aminobutyrate aminotransferase (NM_172961) |  |  | 2.079 |
| ubiquitin specific peptidase 13 (BC090999) |  |  | 2.11 |
| adenylate cyclase activating polypeptide 1 receptor 1 (BC067039) |  |  | 2.111 |
| acetyl-Coenzyme A acyltransferase 2 (BC028901) |  |  | 2.12 |
| glutathione S-transferase (BC044927) |  |  | 2.246 |
| dermatan 4 sulfotransferase 1 (BC043700) |  |  | 2.313 |
| aquaporin 4 (NM_009700) |  |  | 2.317 |
| glucokinase regulatory protein (BC012412) |  |  | 2.34 |
| ATPase (NM_178405) |  |  | 2.515 |
| CDC-like kinase 3 (NM_007713) |  |  | 2.832 |
| mitochondrial creatine kinase (Z13968) |  |  | 2.928 |
| serine (or cysteine) peptidase inhibitor (NM_177920) |  |  | 3.124 |
| Tenascin (X56304) |  |  | 3.561 |
| low density lipoprotein receptor-related protein 2 (XM_130363) |  |  | 6.205 |
| TRIP-Br1 (AF366401) |  |  | 6.205 |
| glia maturation factor-beta (AF297220) |  |  | 6.68 |
| peroxisome proliferator-activated receptor gamma binding protein (AF000294) |  |  | 6.986 |
