# Supplementary material for: Functional cooperation of of IL-1β and RGS4 in the brachial plexus avulsion mediated brain reorganization
Source: J Brachial Plex Peripher Nerve Inj. 2010 Dec 7;5:18. doi: 10.1186/1749-7221-5-18 (PMC3017042; doi:10.1186/1749-7221-5-18)
Supplement: Additional file 3 — Functional classification of the annotated genes that show differentiated expressions in the spinal cord following brachial plexus axotomy. Intensity ratio of cy3 to cy5 was presented for one gene, that was more than 2.0 or less than 0.5 was considered to show prominent up- or down-regulated expression. [file 1749-7221-5-18-S3.DOC]

**Additional file 3**

|  | **1 month** | **3 month** | **6 month** |
| --- | --- | --- | --- |
| **Transcription factors and DNA binding protein** |  |  |  |
| nuclear receptor interacting protein 2 (BC094230) |  | 2.164 |  |
| nucleotide binding protein 1 (BC047926) |  |  | 0.349 |
| zinc finger protein 760 () |  |  | 2.209 |
| nuclear protein (D83033) |  |  | 2.178 |
| **Immune factors** |  |  |  |
| CD72 antigen (BC003824) | 0.48 |  |  |
| interleukin-3 receptor (MUSIL3R) | 3.201 |  |  |
| cdr2 (MMU88588) |  |  | 0.12 |
| dendritic cell C-type lectin protein CIRE (AY049062) |  |  | 0.257 |
| CD177 antigen (BC027283) |  |  | 0.317 |
| neutrophil cytosolic factor 1 (BC055836) |  |  | 0.427 |
| fibrillin 2 (NM_010181) |  |  | 0.487 |
| immunoglobulin superfamily (NM_053199) |  |  | 2.019 |
| growth factor receptor bound protein 2-associated protein 1 (NM_021356) |  |  | 2.025 |
| histocompatibility 2 (BC042572) |  |  | 2.442 |
| **Hormone and transmitter system** |  |  |  |
| methionine adenosyltransferase II (BC058360) | 0.356 |  |  |
| angiopoietin 1 (BC067410) | 2.529 |  |  |
| glucocorticoid receptor (MMGCRR) | 3.251 |  |  |
| adrenergic receptor kinase (BC033272) |  | 3.345 |  |
| thyroid hormone receptor interactor 12 (NM_133975) |  |  | 0.377 |
| **Signal transduction** |  |  |  |
| cationic amino acid transporter (MMU70859) | 0.29 |  |  |
| mitogen activated protein kinase 13 (BC001992) | 0.419 |  |  |
| transmembrane protein 138 (BC058237) | 0.429 |  |  |
| tyrosine phosphatase LAR (AF300943) | 0.452 |  |  |
| mitogen-activated protein kinase kinase kinase kinase 5 (BC048173) | 2.125 |  |  |
| protein tyrosine phosphatase (BC051980) | 2.259 |  | 2.312 |
| cysteine rich transmembrane BMP regulator 1 (NM_015800) | 2.516 |  |  |
| M-cadherin (MUSMCAD) |  | 0.189 |  |
| sphingomyelin phosphodiesterase 3 (BC043077) |  | 0.381 |  |
| protein kinase (BC086660) |  | 2.041 |  |
| ADP-ribosylation factor 3 (NM_007478) |  | 2.106 |  |
| phospholipase C (BC065091) |  | 2.262 |  |
| protein kinase, cAMP dependent regulatory (NM_008923) |  | 2.314 | 2.333 |
| chimerin (BC051139) |  | 2.338 |  |
| vesicle-associated membrane protein (NM_013933) |  | 2.58 |  |
| guanine nucleotide binding protein (BC057665) |  | 2.628 |  |
| regulator of G-protein signaling 4 (NM_009062) |  | 4.23 | 4.64 |
| receptor protein tyrosine phosphatase RPTPmam4 (AF244125) |  |  | 0.237 |
| chloride channel CLIC-like 1 (BC003247) |  |  | 0.286 |
| putative serine/threonine protein kinase MAK-V (AF055919) |  |  | 0.364 |
| ubiquitin-conjugating enzyme E2U (NM_001033773) |  |  | 0.373 |
| transferrin receptor 2 (NM_015799) |  |  | 0.383 |
| RAS related protein 1b (BC033382) |  |  | 0.406 |
| RASD family (BC036988) |  |  | 0.427 |
| mitogen activated protein kinase kinase kinase 7 (BC006665) |  |  | 0.442 |
| tyrosine kinase receptor 1 (BC057004) |  |  | 0.492 |
| protein phosphatase 1 (BC052059) |  |  | 2.02 |
| serine protease inhibitor (BC078639) |  |  | 2.083 |
| FK506 binding protein 5 (NM_010220) |  |  | 2.271 |
| suppression inducing transmembrane adaptor 1 (BC064055) |  |  | 2.756 |
| G protein-coupled receptor kinase-interactor 2 (BC043062) |  |  | 2.991 |
| catenin (cadherin associated protein), beta 1 (BC048153) |  |  | 3.02 |
| serine/arginine-rich protein specific kinase 1 (BC050761) |  |  | 3.748 |
| ubiquitin conjugating enzyme (MMU62483) |  |  | 4.769 |
| ubiquitin protein ligase Nedd-4 (MMU96635) |  |  | 5.243 |
| **Synapse** |  |  |  |
| synaptotagmin XVI (NM_172804) | 2.131 |  | 2.332 |
| synaptobrevin like 1 (NM_011515) | 2.353 |  | 2.543 |
| **Cytoskeleton and motility proteins** |  |  |  |
| tubulin (NM_011655) | 0.456 |  |  |
| kinesin family member 3A (BC052707) | 2.257 |  |  |
| actin related protein 2/3 complex (NM_019824) |  | 0.408 |  |
| kinesin family member 21A (BC060698) |  | 0.478 |  |
| septin 9 (BC046524) |  | 2.105 |  |
| pancortin-3 (D78264) |  |  | 0.423 |
| **Miscellaneous** |  |  |  |
| serine hydrolase protein (MMU245737) | 0.294 |  |  |
| UDP-glucuronosyltransferase (MMUDPGT) | 0.307 |  |  |
| cytochrome P450 (AF336850) | 0.386 |  |  |
| ATPase (NM_178405) | 0.482 |  |  |
| BCL2-associated transcription factor 1 (BC086624) | 0.491 |  |  |
| glutamate-cysteine ligase (BC019374) | 0.498 | 3.304 |  |
| ATP-binding cassette (BC079840) | 2.198 |  |  |
| glycine C-acetyltransferase (NM_013847) | 2.23 |  |  |
| carbohydrate sulfotransferase 12 (NM_021528) | 2.286 |  |  |
| ubiquitin specific peptidase 25 (BC048171) | 2.56 |  |  |
| neuropilin (NRP) and tolloid (TLL)-like 2 (BC068168) | 4.189 |  |  |
| alpha-amylase (MMALP3) |  | 0.212 |  |
| umor protein p53 inducible protein 13 (NM_001024920) |  | 0.214 |  |
| aldehyde dehydrogenase 3 family (NM_026316) |  | 0.281 |  |
| ATPase (BC075718) |  | 0.31 |  |
| laminin B1 subunit 1 (NM_008482) |  | 0.323 |  |
| trypsin 4 (NM_011646) |  | 0.373 |  |
| histone deacetylase 4 (BC066052) |  | 0.42 |  |
| N-methylpurine-DNA glycosylase (NM_010822) |  | 0.433 |  |
| euchromatic histone methyltransferase 1 (NM_172545) |  | 0.488 |  |
| UDP glycosyltransferases 3 family (NM_207216) |  | 0.488 |  |
| Bcl2-associated athanogene 2 (BC016230) |  | 0.491 |  |
| diacylglycerol kinase (NM_138650) |  | 2.142 |  |
| proteaseome (prosome, macropain) 28 subunit (NM_011192) |  | 2.279 |  |
| aspartyl beta-hydroxylase (AF289487) |  | 2.582 |  |
| hexokinase (MUSHEX) |  | 6.945 |  |
| glutaminase 2 (NM_001033264) |  | 8.451 |  |
| monoacylglycerol O-acyltransferase 2 (NM_177448) |  | 15.19 |  |
| mitochondrial tumor suppressor 1 (BC089009) |  |  | 0.02 |
| tripeptidyl peptidase II (BC060627) |  |  | 0.118 |
| TDD5 (MMU52073) |  |  | 0.198 |
| cysteine dioxygenase 1 (BC020375) |  |  | 0.222 |
| ATP synthase (BC048777) |  |  | 0.258 |
| fatty acid desaturase 1 (BC026831) |  |  | 0.313 |
| Bcl6 interacting corepressor (BC058656) |  |  | 0.336 |
| apolipoprotein L (XM_484487) |  |  | 0.394 |
| proteasome (BC014752) |  |  | 0.396 |
| mannose-6-phosphate receptor (NM_010749) |  |  | 0.428 |
| cullin 4B (AY330868) |  |  | 0.442 |
| AN1, ubiquitin-like, homolog (XM_132758) |  |  | 0.461 |
| mitochondrial methionyl-tRNA formyltransferase (BC019509) |  |  | 0.467 |
| low-density lipoprotein receptor-related protein 10 (NM_022993) |  |  | 0.492 |
| protease (prosome, macropain) 26S subunit (BC003860) |  |  | 2.038 |
| CDC-like kinase 3 (NM_007713) |  |  | 2.044 |
| lipoprotein lipase (NM_008509) |  |  | 2.198 |
| glutamic acid decarboxylase 1 (NM_008077) |  |  | 2.245 |
| aldehyde dehydrogenase family 1 (BC054386) |  |  | 2.367 |
| farnesyl diphosphate farnesyl transferase 1 (BC054722) |  |  | 2.416 |
| ATP-binding cassette protein C12 (AF514414) |  |  | 2.621 |
| N-acetyltransferase 6 (NM_019750) |  |  | 2.642 |
| ribonuclease (BC066843) |  |  | 2.743 |
| nuclear mitotic apparatus protein 1 (BC049791) |  |  | 6.435 |
